# Editorial: Education and development in early years from cultural-historical theory

**DOI:** 10.3389/fpsyg.2024.1360576

**Published:** 2024-02-12

**Authors:** Alexander Veraksa, Gustavo Cunha de Araujo, Yulia Solovieva

**Affiliations:** ^1^Faculty of Psychology, Lomonosov Moscow State University, Moscow, Russia; ^2^Federal Scientific Center of Psychological and Multidisciplinary Research, Moscow, Russia; ^3^Faculty of Psychology, Federal University of North Tocantins (UFNT), Araguaína, Brazil; ^4^Faculty of Psychology, Meritorious Autonomous University of Puebla, Puebla, Mexico

**Keywords:** Vygotsky, cultural-historical psychology, executive functions (EF), child development, longitudinal studies

L. S. Vygotsky's cultural-historical approach opened new possibilities for understanding childhood and the ways of acquisition of cultural historical experience during this long period of life, which consists of sequential subperiods and phases (Elkonin, 1995; Vygotsky, 1996; Veraksa, 2022). They can be studied following the identification of periods of child's development introduced by Vygotsky (1983) under the name of psychological ages. Each particular period of psychological development is characterized by its proper way of dominion of cultural experience under the existence of the motive of cultural activity, that is, under active participation in the process of leading activity of each psychological age (Elkonin, 1995).

Vygotsky's provisions on the development of higher mental functions had launched a whole line of research, accomplished by his followers. Neuropsychological research was implemented under the leadership of A. R. Luria. It was aimed at the question of regulation of human behavior by instrumental use of speech and other cultural means, as well as the contribution of brain functional systems to this regulation. In Luria's (1973) views, it is possible to identify the role of frontal lobes in such regulation. However, according to Vygotsky (1982), the psychological process of regulation of activity is based on cultural interaction between adult and child. Nowadays, it is possible to argue that Luria's perspective, in fact, does not reduce this regulation to any brain structure or to some unique psychological process. It rather suggests a gradual interiorization of the means of external instrumental control, which might further pass onto the level of internal mental control (Galperin and Kabilnitskaya, 1974; Solovieva and Quintanar, 2021). This approach to regulation is studied in the work of Rivera Valdez et al..

Current interest in the concept of regulation is related to the construct of “executive functions” proposed in the works of Lezak (1982). In the modern literature, the process of psychological regulation of one's own actions is mostly associated with Vygotsky's understanding of the structure of consciousness and its development (Ardilla, 2013). It happens so, that all aspects of acquisition of self-control, regulation, voluntary activity, and the possibility of conscious regulation of the action is reduced to the unique term of “executive functions.” Gavrilova, Aslanova et al. turned to the assessment of executive functions by educators and revealed the differences in the use of the assessment tool on 5–6 and 7 years old children.

From psychological point of view, the search for opportunities for the development of regulation or self-regulation in children from the perspective of the cultural-historical approach, involves the identification of leading activities (Leontiev, 2000), in which such development occurs most harmoniously and remains sustainable. Mendoza-García and Moreno-Núñez show that an adult plays a key role in child's interaction with objects in early years. The study of play activity, the use of toys and symbols in the preschool age, is in the focus of interest for child development and education experts (Singer, 2015; Solovieva and Garvis, 2018; Solovieva and Quintanar, 2019; Fleer et al., 2020; Gonzáles-Moreno and Solovieva, 2022; Gavrilova et al., 2023). In this regard, Sukhikh et al. explored the emotional side of play as a driver for executive functions' development and provided insights in understanding the mechanisms of play. Bredikyte and Brandisauskiene tested the hypothesis of connection between the development of play and executive functions in children, which again emphasized the developmental potential of play. Gavrilova, Sukhikh et al. sought to analyze the relationship of toy preferences with executive functions' development. These authors found that children, who preferred trendy toys demonstrated a lower level of executive functions. It is of special interest that that, according to their study, trendy toys did not actually serve as a tool for play organization, but could disrupt play activity (Gavrilova, Sukhikh et al.).

The study by Dolgikh et al. revealed that activities different from play could also support executive functions' development and thus, could be included in the education process.

There is no doubt that new social challenges that children are facing in their environment, deserve special attention. Widespread digitalization affects children already in the early years and raises the question of the opportunities and risks that digital devices pose for their development. The need to understand the use of digital devices as objects that can be mastered culturally and support child development, or used naturally, without the involvement of an adult and cultural experience is reflected in multiple research works (Veraksa et al., 2022; Veresov and Veraksa, 2022). Bukhalenkova and Almazova found no relationship between imagination and the time spent by preschoolers on computer games, but revealed a strong connection between imagination and the characteristics of parental participation in the use of digital devices by children. These results outline the importance of an adult in the process of transition of cultural tools. Shatskaya et al. revealed how the use of digital devices could be analyzed from the cultural-historical perspective, and linked to executive functions' development. The results of their study suggest that the use of digital devices in cultural vs. entertaining way starts to dominate in primary school.

As parents report, nowadays, family environment and its key characteristics are also undergoing changes (Sobkin et al., 2013). It is one of the factors that the cultural-historical always considered in childhood research. Yakupova and Suarez discovered a previously uncontemplated relationship between maternal depression and child's emotional development, bringing attention to the emotional state of a parent as an essential aspect of the social situation of child development.

The studies on educational environment, included in this Research Topic, are of particular interest in this regard. In comparison to the family environment, the educational environment demonstrated a greater resistance to change, and retained a critical role in child development because of children's systematic interaction with adults and peers. The contribution of the preschool environment to child development has been of a particular research interest for over two decades (Sylva et al., 2004). At the same time, a number of questions formulated within the framework of cultural-historical psychology require additional research. The potential of such well-elaborated tools as CLASS and ECERS is discussed in the work of Bukhalenkova et al. in the light of Vygotsky's theory. The authors show a balanced approach to combining these tools. Meanwhile, Seo and Song focused on the role of teachers in child development during the pandemic: emotional support from teachers can be a longstanding driver for the child. Finally, Khotinets and Shishova raise an important question of the relationship between the development of creativity and the characteristics of the educational environment.

In his works, Vygotsky laid the foundations for studying the processes of mastering sign systems, in which language plays a primordial role (Luria, 1979). The study of the inextricable connection between the development of speech and higher mental functions determined the formation of a research approach to speech development that, in line with cultural-historical psychology, became classical. Oshchepkova et al. revealed a strong relationship between executive functions and writing skills in children. The obtained results provide valuable practical insights on possibilities of writing skills correction. Tulviste and Tamm demonstrated that the social situation of development, determined by adults, played a key role in the development of children's linguistics skills.

At the same time, the systematization of various means of mastering content (signs, symbols, and models) necessitated the study of the identified conceptual patterns based on mathematics, physics, ecology, geography, and other subjects. Already in early school years, these subjects become important psychological tools in child development. Reflective and conscious mastering of use of cultural conceptual patterns should become a new focus of psychological research in the field of development and learning (Solovieva and Quintanar, 2021). Solovieva et al. describe a training program for mastering basic mathematical concepts in preschool based on Vygotsky's ideas.

The present Research Topic includes relevant methodological considerations and empirical research, related to the cultural-historical theory of child development. The Research Topic contains articles by experts from different countries, which allows investigation of the latest empirical data and its interpretation in cross-cultural aspect.

The Research Topic has opened new ways for the continuation of the discussion of the use of cultural-historical approach in the field of psychological development, and the possibility of a significant modification of the guiding role of adults and social institutions in child development.

## Author contributions

AV: Writing—original draft, Writing—review & editing. GC: Writing—original draft, Writing—review & editing. YS: Writing—original draft, Writing—review & editing.
